# The atrial natriuretic peptide (ANP) knockout mouse does not exhibit the phenotypic features of pre-eclampsia or demonstrate fetal growth restriction

**DOI:** 10.1016/j.placenta.2016.04.003

**Published:** 2016-06

**Authors:** Sarah L. Finn-Sell, Lewis J. Renshall, Elizabeth J. Cowley, Mark R. Dilworth, Mark Wareing, Susan L. Greenwood, Colin P. Sibley, Elizabeth C. Cottrell

**Affiliations:** Maternal and Fetal Health Research Centre, Institute of Human Development, The University of Manchester, Manchester Academic Health Science Centre, St. Mary's Hospital, Manchester, United Kingdom

**Keywords:** Pre-eclampsia, Fetal growth restriction, Atrial natriuretic peptide, Mouse

## Abstract

The ANP knockout mouse is reported to exhibit pregnancy-associated hypertension, proteinuria and impaired placental trophoblast invasion and spiral artery remodeling, key features of pre-eclampsia (PE). We hypothesized that these mice may provide a relevant model of human PE with associated fetal growth restriction (FGR). Here, we investigated pregnancies of ANP wild type (ANP^+/+^), heterozygous (ANP^+/-^) and knockout (ANP^−/-^) mice. Maternal blood pressure did not differ between genotypes (E12.5, E17.5), and fetal weight (E18.5) was unaffected. Placental weight was greater in ANP^−/−^ versus ANP^+/+^ mice. Therefore, in our hands, the ANP model does not express phenotypic features of PE with FGR.

## Highlights

• Mouse models of pre-eclampsia and fetal growth restriction are needed to test potential therapies.
• ANP knockout mice have previously been identified as a potential model of pre-eclampsia.
• We find that these mice do not exhibit pregnancy-associated hypertension, or fetal growth restriction.
• ANP knockout mice do not provide a robust model of pre-eclampsia or fetal growth restriction.

## 1 Introduction

Pre-eclampsia (PE), the leading cause of maternal and perinatal morbidity and mortality worldwide, affects 3–5% of all pregnancies [1]. PE is characterized by the onset of hypertension and proteinuria, from 20 weeks gestation [2], and is associated with placental dysfunction and widespread maternal vascular endothelium activation [3], [4] leading to the pathophysiology of multi-system organ dysfunction. To date, there are no treatments for PE, the only effective intervention being premature delivery of the baby [5], [6], [7].

The development of clinically relevant mouse models of human pregnancy complications such as PE and fetal growth restriction (FGR) is essential for furthering our understanding of these conditions and for testing potential therapies [8]. Despite the fact that, in humans, PE is the leading cause of FGR [9], to our knowledge there remain no reports of a genetically modified mouse that demonstrates PE with FGR.

Atrial natriuretic peptide (ANP) plays a key role in blood pressure regulation and sodium homeostasis [10], [11]. Previous studies have reported that adult male ANP knockout (ANP^−/-^) mice are hypertensive compared with wildtype (ANP^+/+^) counterparts, whilst heterozygous animals (ANP^+/-^) display an intermediate phenotype, becoming hypertensive only after receiving a high salt diet [12]. More recently, Cui et al. demonstrated that adult female ANP^−/−^ mice exhibit pre-pregnancy hypertension, with further systolic blood pressure increases during pregnancy and associated late gestation proteinuria [13]. Pregnant ANP^−/−^ mice also had significantly reduced litter sizes compared with their ANP^+/+^ counterparts, although fetal weight was not reported [13]. Here, we tested the hypothesis that ANP^−/−^ mice exhibit FGR in addition to phenotypic features of PE.

## 2 Methods

### 2.1 Animals

Animal care and experimental procedures were performed in accordance with the U.K. Animals (Scientific Procedures) Act 1986. ANP mice (strain: B6.129P2-*Nppa*^*tm1Unc*^/J), were obtained from Jackson Laboratories and the colony was maintained as heterozygous breeding pairs. Experimental animals of each genotype (ANP^+/+^, ANP^+/−^ and ANP^−/-^) were mated in genotype-matched pairs; the presence of a copulation plug was designated as embryonic day (E)0.5 of pregnancy. Animals had food (Beekay Rat and Mouse Diet; Bantin & Kingman, UK) and water *ad libitum*, and were maintained on a 12:12-h light-dark cycle at 21–23 °C. DNA was extracted from ear clips (offspring, at weaning) or fetal tail tips for genotype determination (primers and PCR conditions available on request).

Blood pressure was measured using a previously validated tail-cuff method (LE5001; Pan Lab, Spain, [14]). Systolic blood pressure (SBP) measurements were made in non-pregnant mice (2–4 days pre-mating) and subsequently in pregnant mice (E12.5 and E17.5). At E18.5, fetuses and placentas were rapidly harvested and wet weights recorded. Fetal weight centile charts were constructed as previously described [15].

### 2.2 Urine albumin and creatinine concentration

Maternal urine was collected at E18.5 by spontaneous voiding and aliquoted and stored at −20 °C. Urinary albumin (mouse ELISA; AssayPro, St Charles, MO), and creatinine (Cayman Chemical Company, Ann Arbour, MI) concentration was measured and the albumin/creatinine ratio calculated.

### 2.3 Statistical analysis

Data are presented as mean ± SD or dot plots of litter means with median. Statistical analysis was by two-way ANOVA or Kruskal-Wallis test as appropriate. *P* < 0.05 was deemed significant.

## 3 Results and discussion

**Fig. 1:**
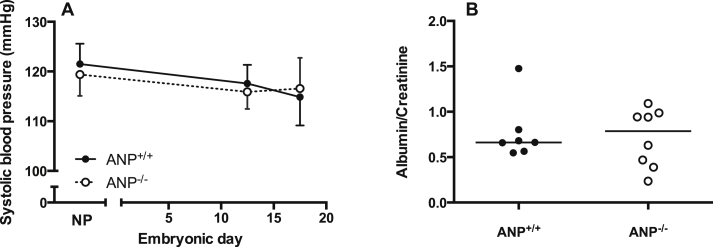
Systolic blood pressure and proteinuria during pregnancy. (A) Systolic blood pressure was not significantly different between ANP^+/+^ (n = 5) and ANP^−/−^ (n = 5) dams either pre-pregnancy (non-pregnant; NP) or at E12.5 or E17.5 by two-way ANOVA. Systolic blood pressure did not increase in pregnancy in either genotype. Data are mean ± SD. (B) ANP^−/−^ mice did not demonstrate proteinuria at E18.5. Lines represent median, n = 7–8.

In contrast to previous reports [13], [16], neither ANP^−/−^, nor ANP^+/−^ dams demonstrated a hypertensive phenotype either before or during pregnancy (Fig 1A). Indeed, the SBP values for all dams obtained in the present study were comparable to that of our C57Bl/6J mice, the ANP model's background strain (E17.5 SBP of 116.5 ± 3.9 mmHg, n = 6). This discrepancy between observations from different laboratories may be due to methodological differences e.g. use of radiotelemetry compared with tail cuff technique employed here. However, it is important to note that the first paper describing the phenotype of ANP^−/−^ animals, which used a similar tail cuff technique to our own, showed only relatively minor differences in SBP unless animals were challenged with a high-salt diet [12]. Furthermore, it is unlikely a deficiency in the tail cuff technique used, as our contemporaneous measurements from pregnant eNOS^−/−^ mice (an established model of hypertension, [17], [18], [19]) demonstrated significantly elevated SBP in this model (E17.5, 136.0 ± 8.7 mmHg, n = 6).

Concomitant with a lack of raised SBP, there was no evidence of proteinuria in ANP^−/−^ compared with ANP^+/+^ dams (Fig 1B). We also found no reduction in litter size from ANP^−/−^ mice (median litter size 7 (range 5–11) pups per litter) compared with litters from ANP^+/−^ and ANP^+/+^ dams (8 (5–10) and 9 (5–11) pups per litter, respectively). However, within our colony we observed fewer ANP^−/−^ offspring surviving to weaning, with only 9.8% (17/174) of total female offspring and 13.5% (23/171) of total male offspring being ANP^−/−^. In contrast, the fetal genotypes in the ANP^+/−^ litters at E18.5 approximated an expected Mendelian ratio (24% ANP^−/−^, 43% ANP^+/−^ and 35% ANP^+/+^). This suggests that the ANP^−/−^ offspring have an increased risk of neonatal mortality, although the reasons for this are currently unknown.

**Fig. 2:**
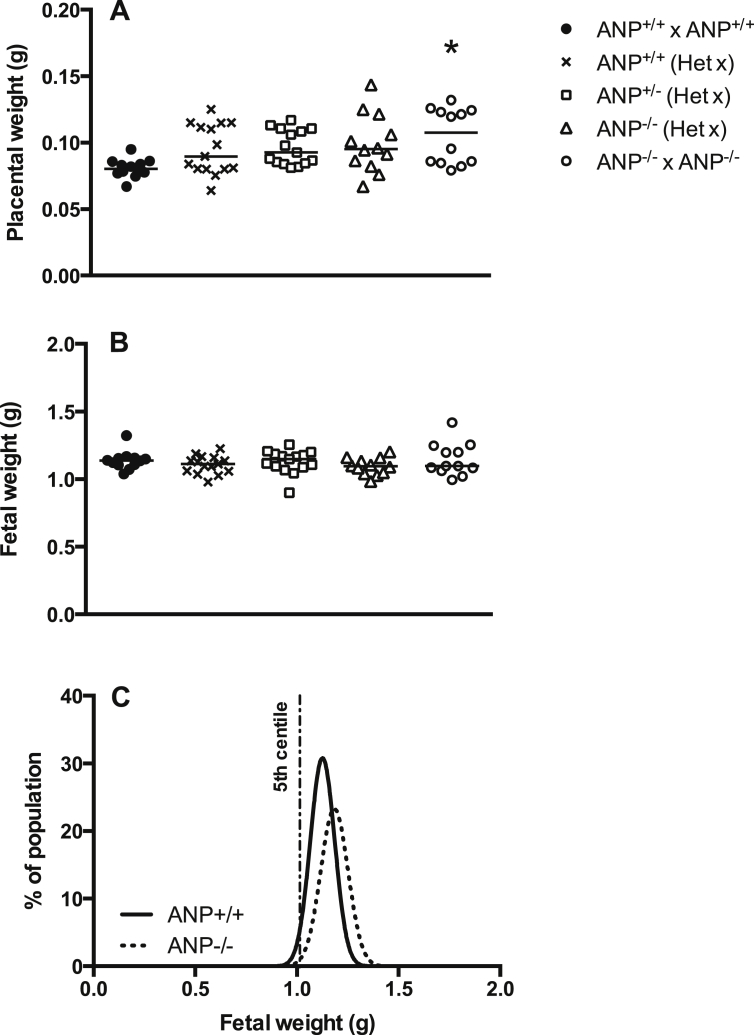
Fetal and placental weight at E18.5. (A) Placental weight was significantly increased in ANP^−/−^ fetuses from ANP^−/−^ x ANP^−/−^ mating pairs compared with ANP^+/+^ from ANP^+/+^ x ANP^+/+^ matings; one-way ANOVA, *P < 0.05. ANP animals of all three genotypes from ANP^+/−^ x ANP^+/−^ matings (Het x) were not different from either ANP^−/−^ x ANP^−/−^ or ANP^+/+^ x ANP^+/+^ offspring. (B) There was no difference in fetal weight between genotypes. Data are litter means (n = 12–15 litters). (C) There was no increase in the number of ANP^−/−^ fetuses falling below the 5th centile of ANP^+/+^ fetal weights (Data from ANP^+/+^ x ANP^+/+^ and ANP^−/−^ x ANP^−/−^ matings, n = 109 ANP^+/+^ n = 75 ANP^−/−^ fetuses.

No differences were observed in fetal weight between ANP^−/−^ (from ANP^−/−^ homozygous matings) and ANP^+/+^ mice (Fig. 2B) and there was no significant difference in the number of fetuses falling below the 5th percentile (defined as FGR, [15]), strengthening the assertion that ANP deletion does not cause FGR. Indeed, there was a non-significant rightward shift of the growth curve of ANP^−/−^ fetuses, which may be related to oedema, which was apparent in a number of fetuses (data not shown). Similarly, placental wet weight was significantly greater in ANP^−/−^ compared to ANP^+/+^ fetuses (Fig. 2A, *P* < 0.01).

In summary, we find that deletion of the ANP gene in mice is associated with increased placental weight and a failure to thrive in the neonatal period, but not with phenotypic features of PE or FGR. These data reinforce the need to identify robust animal models of pregnancy complications in which to test candidate therapeutics, and the importance of fully characterizing animal models in independent laboratories before they can be established for use in obstetric research.

## Conflict of interest statement

The authors confirm no conflict of interest.
